# A Case of Knobloch Syndrome With Lens Dislocation Resembling Homocystinuria

**DOI:** 10.1002/ccr3.72148

**Published:** 2026-02-25

**Authors:** Elnaz Asadollahzadeh, Ali Rezaei, Vahid Shahmaei, Mohammad‐Sadegh Johari, Mohammad Ali Sahraian

**Affiliations:** ^1^ Multiple Sclerosis Research Center, Neuroscience Institute Tehran University of Medical Sciences Tehran Iran; ^2^ MAHAK Hematology Oncology Research Center (MAHAK‐HORC), MAHAK Hospital Shahid Beheshti University of Medical Sciences Tehran Iran; ^3^ Department of Radiology, Faculty of Medicine Aja University of Medical Sciences Tehran Iran

**Keywords:** autosomal recessive, COL18A1, congenital visual impairment, genetic testing, Knobloch syndrome, lens dislocation

## Abstract

We report a 39‐year‐old woman with lifelong visual impairment who presented in June 2024 with progressive visual deterioration in her right eye. Ophthalmologic evaluation revealed severe high myopia, vitreoretinal degeneration, phthisis bulbi of the left eye, and downward lens dislocation of the right eye. Neurological workup revealed bilaterally blurred optic discs, an elevated cerebrospinal fluid opening pressure of 31 cm H_2_O that normalized on repeat lumbar puncture, nonspecific white matter signal changes on MRI, and bilateral frontal polymicrogyria. Initial mild homocysteine elevation prompted consideration of homocystinuria; however, whole‐exome sequencing identified a homozygous frameshift mutation in COL18A1 (c.2824_2831del, p.Gly942Argfs*142), confirming Knobloch syndrome type 1. This case illustrates an adult presentation of Knobloch syndrome with retinitis pigmentosa‐like retinal changes and lens dislocation mimicking homocystinuria.

## Key Clinical Message

Knobloch syndrome (KNO) should be considered in adults with congenital visual impairment, lens dislocation, and retinal degeneration, especially with a positive family history and consanguinity. Differentiation from homocystinuria and Marfan syndrome relies on detailed ocular examination and genetic testing. Early genetic diagnosis guides management and family counseling.

## 1 Introduction

Knobloch syndrome (KNO) is a rare genetic disorder first described by William Hunter Knobloch in 1971 [1]. It is caused by mutations in the COL18A1 gene on chromosome 21, which encodes collagen type XVIII, a protein critical for retinal integrity and neural tube closure. The syndrome is characterized by ocular abnormalities, including retinal degeneration, high myopia, lens dislocation, and occipital skull defects. Due to its rarity, diagnosis is often delayed or misattributed to other conditions [2]. KNO may be mistaken for homocystinuria or Marfan syndrome because of shared features such as lens dislocation, highlighting the need for comprehensive genetic testing [3, 4]. We report a case of KNO in a woman with progressive visual impairment and neurological findings.

## 2 Case History/Examination

A 39‐year‐old woman with congenital visual impairment presented in June 2024 with progressive visual deterioration in her right eye over the preceding months. According to the patient's history, she had complete blindness in the left eye since childhood, while the right eye had reduced but functional vision for many years; complete loss of vision in the right eye occurred only in the months prior to presentation. Her past medical history was notable for long‐standing migraine‐type headaches with a stable pattern and severity. She had no history of systemic diseases, thromboembolic events, developmental delay, or chronic medication use. Family history was significant for migraine‐type headaches and retinitis pigmentosa with blindness in the patient's father. The patient's parents were first cousins (third‐degree consanguinity). No affected siblings were reported.

On physical examination, there were no features suggestive of a marfanoid habitus, including arachnodactyly, joint hypermobility, pectus deformity, or skeletal abnormalities. No systemic findings suggestive of homocystinuria were identified. Ophthalmological examination revealed severe high myopia. The left eye had no light perception, while the right eye had markedly reduced visual acuity. Fundus examination demonstrated posterior staphyloma and peripapillary atrophy, vitreoretinal degeneration with vitreomacular adhesion, macular thinning with cystoid changes, peripheral lattice degeneration predisposing to retinal detachment, and patchy pigmentary changes resembling retinitis pigmentosa with an atypical distribution. Visual evoked potential testing showed bilaterally prolonged P100 latency, consistent with optic pathway dysfunction.

Neurological examination revealed bilaterally blurred optic discs, raising concern for elevated intracranial pressure. Lumbar puncture demonstrated an elevated cerebrospinal fluid (CSF) opening pressure of 31 cm H_2_O (normal range: 7.5–21.1 cm H_2_O in the supine position [5]). Treatment with acetazolamide three times daily was initiated; however, no improvement in visual acuity was observed. Given the lack of clinical response, further ophthalmologic and neuroimaging evaluations were performed. Repeat ophthalmologic assessment suggested a primary genetic ocular disorder, with no evidence of papilledema requiring intervention.

**FIGURE 1:**
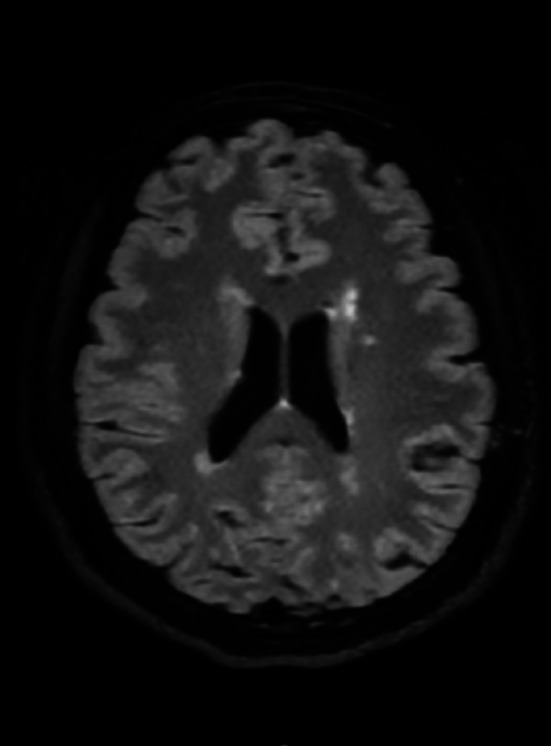
Brain MRI (T2‐FLAIR sequence) showing nonspecific white matter signal changes.

**FIGURE 2:**
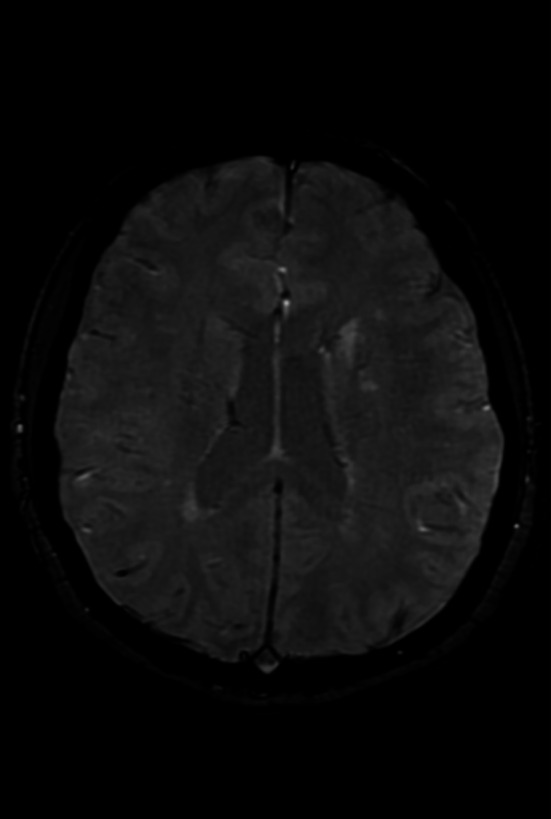
Susceptibility‐weighted imaging (SWI) demonstrates no evidence of central vein sign (CVS) or paramagnetic rim lesions (PRL).

**FIGURE 3:**
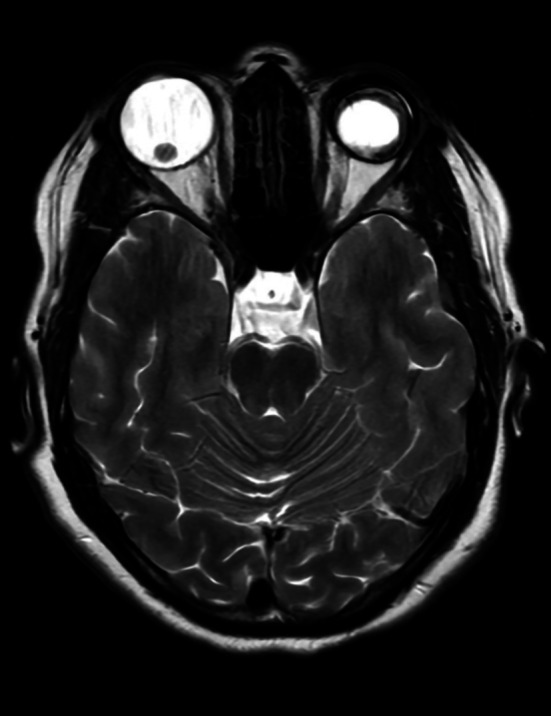
Orbital MRI (T2‐weighted sequence) revealing downward lens dislocation in the right eye and phthisis bulbi in the left eye, characterized by a shrunken, structurally disorganized globe.

**FIGURE 4:**
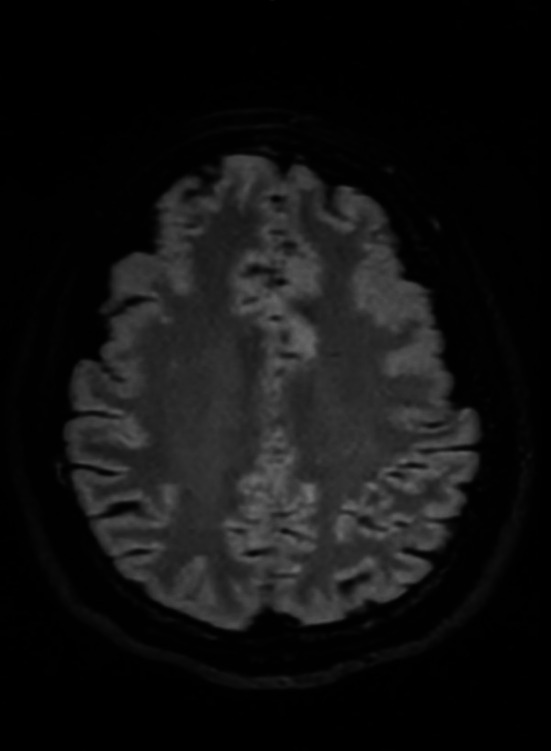
Axial brain MRI (T2‐FLAIR sequence) depicting bilateral frontal polymicrogyria, characterized by abnormal cortical thickening and irregular gyral patterns predominantly affecting the frontal lobes.

Brain MRI revealed nonspecific white matter signal changes (Figure 1), which were attributed to long‐standing migraines. Susceptibility‐weighted imaging (SWI) showed no evidence of central vein sign (CVS) or paramagnetic rim lesions (PRL) (Figure 2). CSF analysis revealed two oligoclonal bands (OCBs) with a negative IgG index. A repeat lumbar puncture demonstrated a normal opening pressure of 12 cm H_2_O. Orbital MRI identified downward lens dislocation in the right eye and phthisis bulbi in the left eye (Figure 3). Brain MRI also demonstrated bilateral frontal polymicrogyria, characterized by abnormal cortical thickening and irregular gyral patterns predominantly involving the frontal lobes (Figure 4).

## 3 Differential Diagnosis, Investigations, and Treatment

Given the downward lens dislocation, homocystinuria was considered in the differential diagnosis. The initial serum homocysteine level was mildly elevated at 12.4 μmol/L (reference range: 4.9–10.9 μmol/L [6]). Acetazolamide was discontinued due to its potential effect on homocysteine metabolism. A repeat homocysteine measurement four weeks later showed a level of 13.34 μmol/L (reference range: 6–14 μmol/L). Whole‐exome sequencing identified a homozygous frameshift mutation in COL18A1 (c.2824_2831del, p.Gly942Argfs*142), confirming the diagnosis of KNO type 1. This variant introduces a premature stop codon consistent with previously reported pathogenic mutations. A chronological timeline of the patient's clinical presentation, diagnostic evaluations, and genetic confirmation is summarized in Table 1.

**TABLE 1:** Chronological timeline of clinical presentation, diagnostic evaluations, and genetic confirmation of the patient.

Time/Event	Clinical findings/actions
Birth	Congenital visual impairment documented
June 2024 (Age 39)	Worsening visual acuity: Right eye further reduced; left eye with no light perception. Neurological examination revealed blurred optic discs. LP: CSF opening pressure 31 cm H_2_O
After initial presentation	Initiation of acetazolamide treatment (TID) with no improvement in visual acuity
Second opinion	Comprehensive ophthalmologic assessment and neuroimaging performed. Brain MRI: Nonspecific white matter changes (attributed to migraines). SWI and CSF analysis ruled out demyelinating disorders
Repeat LP	CSF opening pressure normalized (12 cm H_2_O), indicating no ongoing intracranial hypertension
Ophthalmologic findings	Right eye: Downward lens dislocation. Left eye: Phthisis bulbi
Metabolic evaluation	Serum homocysteine initially 12.4 μmol/L. Acetazolamide discontinued. Repeat homocysteine (4 weeks later): 13.34 μmol/L
Genetic testing	Whole‐exome sequencing identified a homozygous pathogenic COL18A1 mutation (c.2824_2831del, p.Gly942Argfs*142)
Final diagnosis	KNO confirmed, with features overlapping homocystinuria and retinitis pigmentosa‐like manifestations

Abbreviations: μmol/L, micromoles per liter; CSF, cerebrospinal fluid; CVS, central vein sign; LP, lumbar puncture; OCBs, oligoclonal bands; PRL, paramagnetic rim lesions; SWI, susceptibility‐weighted imaging; TID, ter in die (three times daily); KNO, knobloch syndrome.

## 4 Conclusion and Results

Final diagnosis was KNO type 1, confirmed by a homozygous COL18A1 frameshift mutation (c.2824_2831del, p.Gly942Argfs*142). Acetazolamide was discontinued without recovery of vision in the affected eye. Repeat lumbar puncture normalized the opening pressure. The patient was referred for genetic counseling. Ophthalmologic follow‐up was recommended.

## 5 Discussion

KNO is a rare autosomal recessive disorder caused by mutations in the COL18A1 gene, characterized by ocular abnormalities and occipital skull defects. Typical features include high myopia, retinal degeneration, lens dislocation, and occasionally neurological manifestations such as headaches and white matter changes [7]. Our patient presented at age 39, which is unusual, as KNO typically manifests in infancy with ocular problems. Congenital visual impairment since childhood, together with a positive family history of retinitis pigmentosa‐like features and parental consanguinity, supports a genetic etiology and explains the delayed diagnosis until adulthood.

Detailed ophthalmologic examination revealed severe high myopia, vitreoretinal degeneration, and lens dislocation. Left eye phthisis bulbi and downward lens displacement in the right eye initially raised suspicion for homocystinuria. However, mild and stable homocysteine elevation, absence of systemic Marfan features (e.g., tall stature, arachnodactyly, joint hypermobility), and a homozygous pathogenic COL18A1 mutation (c.2824_2831del, p.Gly942Argfs*142) confirmed KNO. This variant introduces a premature stop codon, consistent with previously reported pathogenic mutations. Comparing with previously reported cases, this patient exhibited retinitis pigmentosa‐like retinal changes, expanding the known phenotypic spectrum of KNO [8, 9, 10]. White matter changes on brain MRI were attributed to long‐standing migraines rather than a demyelinating process, supported by negative CSF oligoclonal bands, absence of CVS or PRL on SWI, and normal CSF opening pressure on repeat lumbar puncture, ruling out ongoing intracranial hypertension.

Differentiating KNO from homocystinuria and Marfan syndrome is crucial. Homocystinuria often presents with systemic features, including thromboembolism, fair complexion, and skeletal abnormalities, whereas Marfan syndrome manifests with tall stature, cardiovascular involvement, and skeletal anomalies. KNO diagnosis relies primarily on ocular findings and genetic confirmation.

## Author Contributions

**Elnaz Asadollahzadeh:** conceptualization, writing – original draft. **Ali Rezaei:** writing – original draft, writing – review and editing. **Vahid Shahmaei:** data curation, formal analysis. **Mohammad‐Sadegh Johari:** investigation, validation, visualization. **Mohammad Ali Sahraian:** supervision, visualization.

## Funding

The authors have nothing to report.

## Consent

Written informed consent was obtained from the patient for publication of this case report and any accompanying images.

## Data Availability

No datasets were generated or analyzed during the current study.
